# Family therapy for emerging adults with anorexia nervosa: Expert opinion on evidence, practice considerations, and future directions

**DOI:** 10.1002/erv.3129

**Published:** 2024-08-18

**Authors:** Elizabeth Dodge, Julian Baudinet, Amelia Austin, Ivan Eisler, Daniel Le Grange, Gina Dimitropoulos

**Affiliations:** ^1^ Eating Disorder Outpatient Service South London & Maudsley NHS Foundation Trust London UK; ^2^ Centre for Research in Eating and Weight Disorders (CREW) Institute of Psychiatry Psychology and Neuroscience King's College London London UK; ^3^ Maudsley Centre for Child and Adolescent Eating Disorders South London and Maudsley NHS Foundation Trust London UK; ^4^ The Mathison Centre for Mental Health Research & Education Cumming School of Medicine University of Calgary Calgary Alberta Canada; ^5^ O’Brien Institute for Public Health University of Calgary Calgary Alberta Canada; ^6^ Department of Psychiatry and Behavioral Sciences University of California San Francisco California USA; ^7^ Department of Psychiatry and Behavioral Neuroscience The University of Chicago (Emeritus) Chicago Illinois USA; ^8^ Faculty of Social Work University of Calgary Calgary Alberta Canada; ^9^ Department of Psychiatry Cumming School of Medicine University of Calgary Calgary Alberta Canada

**Keywords:** adolescents_2_, emerging adulthood_4_, family based treatment_7_, transition age youth_5_, young adult_3_

## Abstract

Various forms of eating disorder focused family therapy (FT‐ED) have been developed and evaluated for adolescents. FT‐ED for adolescent anorexia nervosa (AN) focuses on empowering parents/carers with the knowledge and skills required to facilitate recovery for their child. The recent trend and increased awareness of the period between adolescence and adulthood during ages 18–25, known as ‘emerging adulthood’, has brought into question whether the traditional treatment approach for adults with AN, that is, individual therapies, is the most appropriate approach for this age group. This paper briefly outlines the current forms FT‐ED for emerging adults with AN and examines the evidence for each. We then present considerations for tailoring FT‐ED for emerging adults with AN across three broad domains: structure and set‐up, process, and content. Finally, we present suggestions on how to troubleshoot common pitfalls that may be encountered, such as reluctance to include parents in treatment. Future research should examine which forms of FT‐ED are best for which emerging adults and families and under what contexts. There is also a need to explore the incorporation of technology into sessions with families who live apart.

## Highlights

- Multiple forms of eating disorder focused family therapy (FT‐ED) for adolescents with anorexia nervosa have been tailored to an emerging adult population, although no guidelines for these modifications exist.
- Key features of FT‐ED for emerging adults include additional individual time for the emerging adult and greater flexibility in format.
- Troubleshooting strategies to address challenges that develop over the course of therapy, especially hesitance from the emerging adult to include parents/carers, are discussed.

## 1 INTRODUCTION

Emerging adulthood is a life stage that has evolved in the context of current Western culture as an extended period of transition to adulthood. Arnett (2000, 2004) coined the term emerging adulthood to differentiate it as distinct both from adolescence and adulthood. Emerging adults, sometimes also referred to as transition age youth, are young people aged 18–25, although some extend this period up to age 29 (Arnett et al., 2014). This developmental stage is characterised as a time of semi‐autonomy when some, but not all, responsibilities for independent living are taken on. It coincides with changing social and economic circumstances, with a trend for people to remain in the family home well into their 20s. Emerging adulthood is a time of ongoing identity formation when emerging adults do not see themselves as adolescents but often also do not feel entirely adult (Arnett et al., 2014). For many, the period of emerging adulthood is experienced positively, however for others, the need to negotiate the multiple transitions of emerging adulthood without the regular support of family increases vulnerability, which can be associated with eating disorders (EDs) and other mental health problems (Potterton et al., 2020; Tanner, 2016).

Emerging adults with EDs face several challenges (Potterton et al., 2020). The division of child and adolescent from adult mental health services is still the model in Western healthcare systems, despite positive evidence for psychological services across this age division (McGorry et al., 2022). Age related transitions frequently lead to a change of treatment model and different expectations placed on family members, disrupting continuity of care, leading to gaps in treatment and potential setbacks in recovery (Winston et al., 2023). This divide has likely played a part in preventing more developmentally appropriate interventions for emerging adults. Those who are already receiving treatment from child/adolescent ED services may also be navigating a change in treatment provider, either through a move to adult ED services and/or to a university setting (Dimitropoulos et al., 2018, 2019; Dimitropoulos, Freeman, et al., 2015; Potterton et al., 2020).

Historically, the recommended treatment for emerging adults with anorexia nervosa (AN), as for all adults, has been individual therapy with support for families/carers often delivered in separate workshops or group format (Hannah et al., 2022). These carer skills workshops have been developed and disseminated widely across adult ED services (Langley et al., 2018). However, the recent American Psychiatric Association (APA) Guidelines (Crone et al., 2023) now suggest more extensive family involvement for emerging adults, recommending ‘*that adolescents and emerging adults with AN who have an involved caregiver be treated with ED‐focused family based treatment, which should include caregiver education aimed at normalising eating and weight control behaviours and restoring weight*’ (p.168)

Despite this recommendation, there is no consensus as to what constitutes effective inclusion of family members in the treatment of emerging adults with EDs. For this age group, most evaluative appraisals of treatment options that involve families focus on the support of carers as an adjunct to individual care, with few examples of conjoint interventions incorporating both the individual and family members (Fleming et al., 2021). These conjoint interventions include the Maudsley Model of AN Treatment for Adults (MANTRA; Schmidt et al., 2023), an individual therapy that includes parents/carers in the collaborative development of the formulation and in at least two sessions during treatment (Schmidt et al., 2015), as well as the First Episode Rapid Intervention for Eating Disorders (FREED) care pathway, which places a strong emphasis on family involvement while delivering any individual, evidence‐based therapy (Allen et al., 2020). While these individual approaches are inclusive of family, the aim of this paper is to examine therapy approaches that are primarily family oriented by briefly presenting the current evidence, delving into practice considerations, and suggesting avenues forward.

### 1.1 Family interventions for emerging adults

There has been a breadth of research evaluating the benefits and challenges of family therapies for emerging adults. For the purposes of this paper, the umbrella term ED focused family therapy (FT‐ED) will be used to describe a variety of single‐family and multi‐family interventions. It encompasses several related forms of intervention, including Family‐Based Treatment (FBT; Lock & Le Grange, 2012), Parent‐Focused Therapy (PFT; Le Grange et al., 2016), Maudsley Family Therapy for AN (FT‐AN; Eisler, Simic, Blessit, et al., 2016) and multi‐family therapy (MFT; Simic et al., 2021). While some differences between these models exist, they are more similar than different (Gorrell et al., 2023). What predominantly unites them is that they all emphasise the importance of the family as a resource to the emerging adult in treatment with a greater focus on strengths, rather than possible causal factors of the illness. FT‐ED treatments are regarded as the first‐line treatment for adolescent EDs (Crone et al., 2023; NICE, 2017).

#### 1.1.1 Single‐family interventions

Outcome data for eating disorder focused single family interventions for emerging adults has been reported in four studies (Chen et al., 2016; Dimitropoulos et al., 2018, 2019; Nyman‐Carlsson et al., 2020). Dimitropoulos et al. (2018, 2019) reported that 25 sessions of FBT for transition aged youth (FBT‐TAY), an adaptation of adolescent FBT, is feasible and associated with weight restoration and improvements in ED symptoms, broader psychological functioning and parental self‐efficacy. Adaptations include prioritisation of the emerging adult's concerns and challenges related to their age group, such as identity development, educational or vocational aspirations, relationships and future goals. Qualitative data on FBT‐TAY emphasises the importance of therapist flexibility, collaboration with parents and carers, addressing developmental issues in treatment planning and delivery, and emphasising the discomfort and temporary nature of increased parental support (Dimitropoulos, Freeman, et al., 2015).

Similarly, Chen et al. (2016) tested the acceptability and efficacy of 6‐month of an adapted FBT for weight restoration in young adults (FBTY) aged 18–26 years with AN. In this open trial, four adaptations were made; (1) young people had the opportunity to choose friends or roommates as their support adult, (2) therapists implemented a more collaborative approach, (3) therapists addressed specific developmental issues, and (4) therapist offered the option of adding additional individual sessions in the later stages of treatment. The study found that most participants chose a parent as their support person, with weight restoration comparable to adolescent data. Of note, they reported relatively high dropout rates and a shorter‐than‐expected treatment length (Chen et al., 2016).

More recently, Nyman‐Carlsson et al. (2020) conducted the only randomised control trial (RCT) comparing individual cognitive behavioural therapy (CBT) for young adults versus combined family/individual therapy for young adults (FT‐YA). Both treatments lasted 18 months, were adapted for emerging adults, and manualised, with the FT‐YA manual (Paulson‐Karlsson et al., 2013) based on the FBT manual for adolescents (Lock & Le Grange, 2012). The main adaptations were to offer a higher number of individual sessions and to exclude the family meal session. Both treatments appeared to be beneficial in terms of weight restoration and reduced ED and general psychopathology. Despite outcomes being similar in both treatments, the authors noted that almost 60% of participants in the FT‐YA group moved out of home during the study. The authors suggested that families may not need to be living together to fully benefit from a family intervention (Nyman‐Carlsson et al., 2020).

#### 1.1.2 Multi‐family therapy (MFT) interventions

Another area of promising family‐informed treatment has been MFT. In this treatment approach, several families come together to form a group and engage in therapeutic activities facilitated by one or more therapists (Baudinet et al., 2021a; Baudinet & Eisler, 2024). Specific MFT models have been developed for adolescent AN (Simic et al., 2021) and emerging adults (Knatz Peck et al., 2021; Skarbø & Balmbra, 2020; Tantillo et al., 2020). Adolescent models build upon the principles of FT‐AN (Eisler, Simic, Blessitt, et al., 2016), and extend to include greater treatment intensity, peer support and the voice of lived experience in treatment (Baudinet et al., 2021a; Dawson et al., 2018).

MFT models that focus on the specific needs of emerging adults are generally more heterogeneous compared to adolescent models. Outpatient adult MFT treatments described in the literature include a stand‐alone intensive 5‐day model (Knatz Peck et al., 2021), a 26‐week model (Tantillo et al., 2019) and a 12‐month model (Skarbø & Balmbra, 2020). MFT for adults has also been used in inpatient care (Coopey & Johnson, 2022) and day patient treatment (Dimitropoulos, Farquhar, et al., 2015).

The only outpatient RCT in MFT recruited adolescents and emerging adults (age 13–20) and compared FT‐AN with and without the addition of 10 days of MFT (Eisler, Simic, Hodsoll, et al., 2016). Participants in the MFT arm had significantly improved outcomes compared to FT‐AN alone, and age was not a treatment moderator (Baudinet, Hodsoll, et al., 2023), suggesting both interventions can be helpful for emerging adults. Quantitative data from an intensive 5‐day temperamentally‐informed MFT for emerging adults indicates it can lead to significant improvements in ED symptoms, body mass index, anxiety/depression, and overall psychological distress (Knatz Peck et al., 2021).

MFT offers additional benefits beyond symptom reduction. It appears to help families develop a better understanding of the potential maintenance factors for the ED, such as interpersonal difficulties, bio‐temperamental factors, family dynamics, and emotional difficulties (Baudinet, Eisler, et al., 2023; Baudinet et al., 2024; Baudinet et al., 2021a; Coopey & Johnson, 2022; Knatz Peck et al., 2021). Qualitative findings from emerging adults indicate that MFT can help them feel better able to express their voice and feel empowered to grow their independence by exploring the impact of ED on their self‐development (Brinchmann & Krvavac, 2021; Coopey & Johnson, 2022). Participants expressed that the experience of connecting with fellow group members led to more openness and in turn, improved communication within their family.

#### 1.1.3 The current dilemma

Overall, there are indications that FT‐ED for emerging adults with EDs is associated with improvements in physical and psychological well‐being. As of yet, it remains unclear how to optimally include families in emerging adults' treatment. In the section below we aim to outline current considerations and guidance to clinicians who are offering FT‐ED to emerging adults with AN.

## 2 PRACTICE RECOMMENDATIONS AND CONSIDERATIONS

Available theory and data point to a few important areas for consideration when implementing and adapting FT‐ED for emerging adults. Practice implications fall under three broad inter‐related domains; considerations for structure and set‐up, process, and content. Structure and set‐up refer to the way treatment is introduced to families, explicitly discussing length and expectations of treatment, collaborative goal setting, session frequency, who attends, what peoples' roles might be and what outcomes can be expected. Process refers to *the way* in which sessions are facilitated whereas content refers to what gets talked about in sessions. Each influence the others and are considered below.

### 2.1 Structure and set‐up

#### 2.1.1 Who to refer?

Prior to the recent APA Guidelines (Crone et al., 2023) FT‐ED has not been a first‐line recommended treatment for people aged 18 years and older. Given similar outcomes between FT‐ED and CBT in the only emerging adult RCT, it is reasonable to consider and/or offer FT‐ED as an equivalent choice for emerging adults who (a) still live at home, (b) have expressed a desire for family/carer involvement and support, and (c) have a relatively short duration of illness. Of note, the only available data currently is for those presenting with AN and related restrictive EDs.

FT‐ED might be contra‐indicated when there has been previous experience of FT‐ED with limited or minimal response and/or the presence of current or historic domestic violence. Notably, these considerations are preliminary and based on clinical experience, and still need to be systematically and empirically tested.

#### 2.1.2 Comprehensive assessment

FT‐ED for emerging adults requires the assessing clinician to be ‘thinking about the entire family’ prior to assessment both in terms of who is invited and how the assessment is undertaken. This will help to open up opportunities for exploring what family intervention may be optimal for the emerging adult and their parents/carers. FT‐ED places primacy on the establishment of collaborative treatment goals by fostering relationships where the emerging adult ‘invites’ support from parents and other supportive individuals in their lives. The therapist should focus on establishing trust and transparency between the emerging adult and parents/supportive others in order that a balance for responsibility for illness management can be achieved. Age‐appropriate boundaries will also need to be set around confidentiality and when these may need to be redefined for example, where there is risk. At times, this may look similar to the set‐up undertaken with older adolescents. However, when working with emerging adults the therapist must be mindful of the legal position, confidentiality and engagement with the young person specifically.

The initial assessment session is particularly important. In addition to understanding the course of the ED, its onset, nodal points and impact, this is the first opportunity to engage the family. Ensuring there is time specifically for the emerging adult alone sends a clear message that they will not be lost in the FT‐ED process, that they have a voice and that the multi‐disciplinary team is not only expecting but will ensure their ongoing contribution to the treatment process. It is also respectful of their developmental stage, regardless of whether they want more independence or are more uncertain about it. Having said that, time with parents and as a whole family is also offered. Again, this sends a clear message that everyone has an important role in treatment. The assessment should also include psychoeducation about emerging adulthood as a developmental stage. This helps family members to connect with current transitions and stressors and will be useful as treatment progresses if/when distress increases or people feel less motivated or hopeless/helpless. It can be helpful to explicitly state the types of situations where parents may need to do more than they might expect at this age and developmental stage (e.g., complete food refusal, consistent weight loss, etc).

### 2.2 Process

#### 2.2.1 Individual time and greater flexibility with format

The first adaptation is to ensure the emerging adult with AN has more individual time with the therapist than is typically offered in FT‐ED for adolescents. Not all emerging adults will want this, in which case it does not need to be insisted upon nor required, however, offering additional time routinely from the outset (including assessment) will send a clear message that it is developmentally appropriate to discuss individual needs away from the family. This may be time within the session in addition to separate individual sessions and may increase as symptoms remit. Parents also can be seen separately with clear expectations about confidentially, for example, that knowledge about symptoms must be brought to treatment with the emerging adult.

Additionally, working with emerging adults may involve clinicians being more flexible with treatment format. For instance, if the emerging adult needs to relocate or travel, explicitly offering and accommodating people joining remotely from different locations may be an age‐appropriate way of including the family while allowing the emerging adult to engage in their broader life in developmentally appropriate ways. In the post‐COVID‐19‐pandemic world, this has become increasingly accessible to all, not just emerging adults. While this is the case across the age range, this is particularly pertinent to emerging adults where the likelihood of moving away for post‐secondary education or out of the family home is more common.

#### 2.2.2 Clarity around carer involvement, respective roles and information sharing

One of the biggest challenges about accepting familial support for emerging adults is the uncertainty about who takes responsibility for illness management (Dimitropoulos et al., 2016). This is a dilemma for emerging adults who are unwell with an ED (or any illness) and may need support but may also desire increased autonomy. It is important for a clinician to name this dilemma at the outset and support age‐appropriate negotiation about each person's level of responsibility. One example of this may be how and when weight is shared between the emerging adult and parents. These conversations are typically driven by the principle *‘the emerging adult* *can* *lead in the recovery process and decision making unless progress stalls or their health deteriorates, at which point carers do more to* *support* *.’* The key difference when working with emerging adults is that this is always based on collaboratively agreed consent which the emerging adult or carer can withdraw at any point if they think it is no longer working. The process of negotiating the parents' role in treatment is often more important than what is actually agreed. Indeed, there will be some emerging adults for whom the support from parents may be more symbolic than real.

In most countries, once someone turns 18, they have the legal right to refuse treatment and to dictate who attends treatment sessions. This is true of any treatment, regardless of illness type or treatment modality. This can be further complicated within the realm of EDs when the individual is very unwell, there is a voiced desire to remain unwell and motivation is low. If an individual is actively refusing treatment and where there is a high level of risk, then compulsory treatment will need to be considered and family members would be involved in accordance with the relevant guidelines. Where there is agreement for therapy to continue, consent for carer involvement will be continuously negotiated and revisited. It is important that this is framed as enhanced support, rather than carers taking ‘control’ over certain aspects of the emerging adult's life, unless they have explicitly consented to this happening for a brief period of time to support the renourishment process. This may require regularly revisiting the pros and cons of carer involvement, a renegotiation of what level of involvement they have and a willingness from the individual to revisit involvement in the future if consent is revoked. From clinical experience, in many circumstances involving parents/carers in treatment can also enhance engagement.

#### 2.2.3 Who's in the room: Is it family or ‘network’ therapy?

The question of who makes up the ‘family’ in FT‐ED is not always simple to answer. As emerging adults move out, begin to become more socially and sexually active, and begin employment, the nature of their support system changes. Although the guiding principle behind involvement of the ‘family’ in FT‐ED is to include significant others to build upon existing strengths, considerable thought from the clinician is needed as to how to avoid creating inappropriate responsibility. Partners, close peers, and other significant figures might attend once or a small number of times. This can be negotiated throughout treatment based on changing need, risk and level of independence. It may also be helpful to further explore possible appropriate roles of non‐parent adults (e.g., adult siblings). While significant others beyond parents are often included in sessions, it is still most common for parents/carers to take the most responsibility out of the wide range of people who may attend.

### 2.3 Content

#### 2.3.1 Engagement and the early stages of treatment

It is likely that engagement will need to be longer than one might expect with family treatments for child and adolescent EDs. The additional tasks such as negotiating roles, identifying the support network, formulating collaboratively and building trust will likely need additional time. This is not always easy, as there is likely urgency to return to a physically healthy state if the person is underweight or physically compromised. Given data that early weight gain is predictive of treatment response (Hamadi & Holliday, 2020), it is important to stay focused on improving health *and* ensuring that the establishment of a trusting relationship is not rushed.

The initial sessions are, therefore, more exploratory and include explicit discussion, often separately from parents, about what would need to be different for the emerging adult to be able to accept parental help. Additionally, it is important to consider the impact of relational issues on the formulation with a greater focus on the tension between needing increased parental/carer support, whilst also being at a life stage that typically expects more independence. See Baudinet et al. (2021b) for a discussion on how to formulate in FT‐ED.

For many emerging adults, this can be a difficult phase of treatment, moving between the desire to be independent and needing a high level of support. When motivation is low and consent for carer involvement is fragile, the therapist might choose to frame a trial of increased carer involvement (or independence) as a time‐limited experiment, agreeing on a time frame, tasks to be managed within this time and what will happen if these tasks are not accomplished.

#### 2.3.2 Middle phase of treatment

One central difference between FT‐ED with emerging adults compared to adolescents is that the phase of treatment focused on symptom management and weight restoration is shorter. This matches the expectation of the emerging adult having greater responsibility for change within treatment (and in life more generally). By intentionally shifting responsibility for continued weight gain and symptom management earlier in treatment, the clinician is sending a clear message about the temporary nature of parental involvement required only when the emerging adult's health is compromised. While weight restoration may still take some time, the responsibility for weight restoration and symptom management is shifted earlier to the young adult, as compared to children and adolescents.

During this phase there is more variability in the roles of family members, which may also change over time. For example, monitoring of weight is mostly between patient and therapist unless agreed otherwise. The parents' role is more likely to be framed as providing support, which may include preparing food, acting as a ‘sounding board’ for food choices, or just being around at mealtimes rather than supervising. Similarly, there is likely to be more flexibility with variety, timing and setting of meals, mainly driven by the emerging adult.

Once the emerging adult has established a steady weight gain trajectory with the support of their family (rather than necessarily reaching a healthy weight), the clinician can usually move to the next phase, that is, discussing broader life issues including the role of AN in the management of emotions and interpersonal relationships. This will typically start with conversations about how much support the emerging adult needs from parents. At this point, there is more of a focus on individual time, even though treatment content will still include questions around family relationships, managing emotions and vulnerabilities in a family context. The content of individual sessions here is to understand the individual's current goals, aspirations, challenges, support needs and experiences of receiving support. There is also an important focus on ‘reclaiming responsibility’. This may include tolerating greater ‘lapses’ with a focus towards the later parts of treatment on supporting the emerging adult to problem solve these independently.

#### 2.3.3 Ending treatment

Typically, this is a time when more conjoint family sessions may recommence, as a way of punctuating the termination of treatment, reviewing each individual's role in recovery and planning for times of stress and possible lapse/relapse ahead. One possible pitfall is that re‐joining at this time can prolong/restart treatment because of anxiety about completing a treatment that has been beneficial to their family. Relapse prevention sessions should be brief and clearly focused on termination of treatment. The tone of these sessions is that any slip is the responsibility of the emerging adult, with discussion about what parents should do in the face of such circumstances.

## 3 ADDRESSING CHALLENGES IN THE COURSE OF THERAPY

### 3.1 The emerging adult does not want any family members or carers involved

This is a common dilemma for anybody working with emerging adults. Given the often‐typical practice of individual work in adult services, it can be easy to quickly abandon the idea of family involvement and move back to an individual approach. This resistance might be particularly strong for emerging adults who have previously attended FT‐ED in child/adolescent services. However, it is important to tease apart how much of this resistance is a desire for independence and how much is due to other concerns, for example, fear of weight gain/being controlled by parents, feelings of shame, avoidance of unpleasant conflicts, or not wanting to continue to be a burden. When faced with resistance to family involvement, rather than insisting, spend time exploring. Increase support for the emerging adult in a way that feels helpful, rather than an enforcement of parental involvement. This is also a time to be curious about other potential supports who might be included.

### 3.2 The emerging adult never wants individual time

It can be easy for a clinician to just see the whole family every session, but this might unintentionally block the emerging adult from moving towards more developmentally appropriate independence. The clinician's role is to explore and gain a systemic understanding of what is happening. Rather than assume this resistance is a problem, explore the concerns regarding separate sessions from the parents as well as the emerging adult.

### 3.3 Parents feel it is inappropriate to be involved

While it is appropriate for parents to expect their emerging adult to be more independent in all aspects of life in comparison to an adolescent, this is an opportunity to recognise how much the illness might be ‘keeping them back’. The focus and tone of these conversations is on the jointly negotiated temporary nature of increased parental support, as well as their attachment desire to care for their child. However, the clinician may conclude that active parental involvement in treatment may be unhelpful and that individual treatment is the best way forward.

When thinking about engaging other family members it is important to be mindful of recognising differences in beliefs between or within families. Family narratives about EDs or emerging adulthood may be shaped by the family's cultural background, history, and experiences. It is important to explore with the parents the reasons for hesitancy to take part in treatment. They may fear encroaching on the emerging adult's sense of autonomy, want to protect their family from shame and stigma, or seek to avoid potential conflict. A sensitive exploration of these issues is important to recognise and validate the possible reasons for ambivalence.

### 3.4 Progress has stalled and the emerging adult no longer wants parental involvement

This is not uncommon and is often a sign of increased anxiety. It can be useful for the clinician to pause and remind the emerging adult of the initial commitment to parental involvement. It can also be useful to frame parents/carers re‐joining treatment initially for a review so that everyone can be updated and a safe plan for treatment can be made. When discussing the need for other people to be involved, it can be useful to consider the upcoming months with/without the additional support, as well as the common paradox of AN—that increased restriction often results in reduced autonomy. This can then be important to link with future life goals explored at the beginning of engagement.

### 3.5 Caregiver fatigue

It is important to acknowledge that, just as the young adult has a choice whether parents should be involved, parents have a choice not to be involved. One of the drivers of parental withdrawal in treatment is fatigue. This is especially true for carers who have been involved in prior treatment with their loved one during adolescence. For some young people and carers, family treatment can feel exhausting and disempowering (Conti et al., 2021; Wufong et al., 2019). The clinician will need to constantly review with all family members where they can be most effective with given resources. It may be negotiated that separated sessions, or less frequent carer involvement for a period of time, may be needed. The clinician may explore with carers the need to access their own support.

## 4 DISCUSSION

This paper aimed to provide a brief update on the current evidence base of FT‐ED for emerging adults, as well as practice considerations and future directions. Increasingly, there is a move towards greater involvement of carers in ED treatment beyond adolescence (Crone et al., 2023). Data are only beginning to emerge regarding the efficacy of interventions for this age group, although what has been reported is promising. Nevertheless, uncertainty remains regarding the best way to involve significant others in treatment and how best to navigate the developmental needs of emerging adults in family treatments.

Specific considerations for emerging adults include increased flexibility as to who attends, when they attend and their role in treatment. Typically, the emerging adult will lead treatment more than would be expected in child and adolescent FT‐ED, with carers typically taking on a more supportive role. In depth discussion around consent to family involvement is needed, and treatment content is much more focused on the challenges of emerging adulthood, such as independent living, employment, romantic relationships, etc. Carers are invited to support the emerging adult in their decision making and provide age‐appropriate guidance.

The considerations presented in this paper are not unique to FT‐ED. As mentioned previously, stand‐alone carer interventions for adults with EDs (Langley et al., 2018) are already routinely being offered by many services. Carer support is already realised in other adult ED treatments, such as MANTRA (Schmidt et al., 2023), CBT‐E (Dalle Grave & Pike, 2023; Fairburn, 2008), and specialist supportive clinical management (McIntosh et al., 2023). Some of these treatments, such as MANTRA, already recommend carer involvement. In MANTRA, a broader definition of carer may be used to include significant others, for example, siblings, friends, parents or partners. In clinical trials, the inclusion of two sessions with a carer or several carers was recommended (Schmidt et al., 2015). In clinical practice the number and timing of carer sessions in MANTRA can vary and is dependent on a range of factors. With younger adults, particularly those who are transitioning from child/adolescent services, involving carers for several sessions at the start of the transition can be helpful to enable the best possible opportunities for engagement (Schmidt et al., 2023). Here, patient and therapist would agree what will be explored before the session and could include sharing of the formulation, support that can be offered for the emerging adult to move forward and looking at ways to address any stuckness or manage deterioration (Schmidt et al., 2023; Wittek et al., 2021, 2023). This approach can also be adopted where emerging adults enter treatment without transitioning from child/adolescent services.

Given the unique demands of emerging adulthood, the authors encourage clinicians, regardless of the treatment model being used, to consider the collaborative and developmentally appropriate inclusion of family members and significant others into treatment. Empirical evaluation is needed to determine the impact on outcome and service satisfaction.

## 5 CONCLUSION

Family therapy is an understudied, yet potentially useful intervention for emerging adults. Various models of FT‐ED have been piloted, but future research is needed to identify which models work best for whom and under what circumstances. High quality outcome data are now needed to confirm the effectiveness of family approaches for this group. A systematic review of available data is also needed.

## CONFLICT OF INTEREST STATEMENT

JB and IE receive royalties from Routledge for a published manual for Multi‐family Therapy for AN (Simic et al., 2021).

## PATIENT CONSENT

Not applicable.

## PERMISSION TO REPRODUCE MATERIALS

Not applicable.

## Data Availability

Data sharing is not applicable to this article as no new data were created or analyzed in this study.
